# Vulnerability of the HIV cascade of care in an Amazonian town: A qualitative study

**DOI:** 10.3389/fpubh.2022.1110330

**Published:** 2023-01-25

**Authors:** Flavia Divino, Mathieu Nacher, Rafael dos Santos Pereira, Paulo Peiter

**Affiliations:** ^1^Laboratório de Doenças Parasitarias, Instituto Oswaldo Cruz, Fundação Oswaldo Cruz, Rio de Janeiro, Brazil; ^2^Centre d'Investigation Clinique Antilles–Guyane (Inserm CIC 1424) Pôle Guyane, Université de Guyane, Cayenne, French Guiana

**Keywords:** HIV, AIDS, public health, Oiapoque, Amazonian town

## Abstract

The HIV care cascade spans from diagnosis to patient linkage and retention in health services for treatment. Brazil has made substantial efforts to optimize the cascade of care. However, despite these advances, there are striking regional differences and difficulties from testing to treatment, particularly in the north and northeast regions, often reflecting social inequalities. Oiapoque, a highly affected city in the state of Amapá, shares its borders with an overseas European territory—French Guiana. The objective of this study was to get a clearer picture of the different components of the HIV care cascade in the municipality of Oiapoque. The study was exploratory and qualitative, involving the mapping of health structures in the research area and interviews with the responsible healthcare professionals working in the municipality. Patients are vulnerable at several levels, including mobility limitations, mismatched information that affects the linkage and retention of treatment, an absence of infectious disease doctors, an absence of user autonomy, missed appointments, dropouts, and abandonment of care. We found that the five recommended steps in the continuum of care for people living with HIV all had weak points or were non-existent or unavailable. These results will be fundamental to rethink the municipality's actions and the strategies of the Unified Health System SUS for the HIV epidemic in these border regions of the Amazon.

## 1. Introduction

Brazil has been at the forefront of the fight against AIDS and HIV worldwide. Brazil makes treatment and testing freely available, was one of the first countries to provide self-testing, challenged pharmaceutical companies in the 1990's for updated versions of antiretroviral drugs, and buys and distributes the most condoms, and in 2013, it began providing free treatment to all individuals living with HIV. Nationwide, the system aimed to test, link, and retain patients to health services, support adherence to antiretroviral therapy (ART), and reinsert those who for some reason had interrupted care (1). The HIV care continuum, or cascade of care, is a public health model that outlines the steps that people with HIV go through from diagnosis to achieving and maintaining viral suppression to restore the immune system and prevent transmission (undetectable = untransmissible). The steps of this cascade are as follows: diagnosis of HIV infection, linkage to HIV medical care, reception of HIV medical care, retention in medical care, and achievement and maintenance of viral suppression. This model also guides the HIV program in Brazil. However, despite improved access to information and social participation, there are unfortunately large regional differences, reflecting persistent social inequalities (2). Significant regional differences are noted by published federal reports: the gross domestic product per capita is substantially lower in the northern states of Brazil than in the southern states, the medical density in the north and the number of public and private hospitals are the lowest in Brazil, and life expectancy at birth is 4–5 years shorter in the north relative to the richer southern states (2).

Throughout Brazil, there has been a stabilization in the AIDS incidence rate in the last 10 years, registering an average of 20.7 new cases per 100,000 inhabitants. There are, however, important regional differences that must be considered. Between 2006 and 2015, there was an important drop in the incidence rate of 23.4% in the southeast region, a drop of 7.4% in the south region, and a stabilization of the rate in the midwest region. In contrast, in the same period, there was a 37.2% increase in the incidence rate in the northeast region and 61.4% in the north (3). The epidemiological bulletin still highlights the following five states in the north and northeast regions that continue to show an increase: Sergipe (23.1%), 52 Alagoas (18.5%), Rio Grande do Norte (9.7%), Amapá (1.7%), and Paraíba (1.5%) (4).

In June 2021, about 1,045,355 cases of AIDS had been reported in Brazil since the first notification of the disease in 1982 (4). Despite a decline in the number of deaths following improved treatment coverage and adherence, the northern and northeastern regions of Brazil still face an active HIV epidemic. In a recent 5-year period (2015–2019), the northern region of Brazil averaged 4,400 cases per year (4).

**Figure 1:**
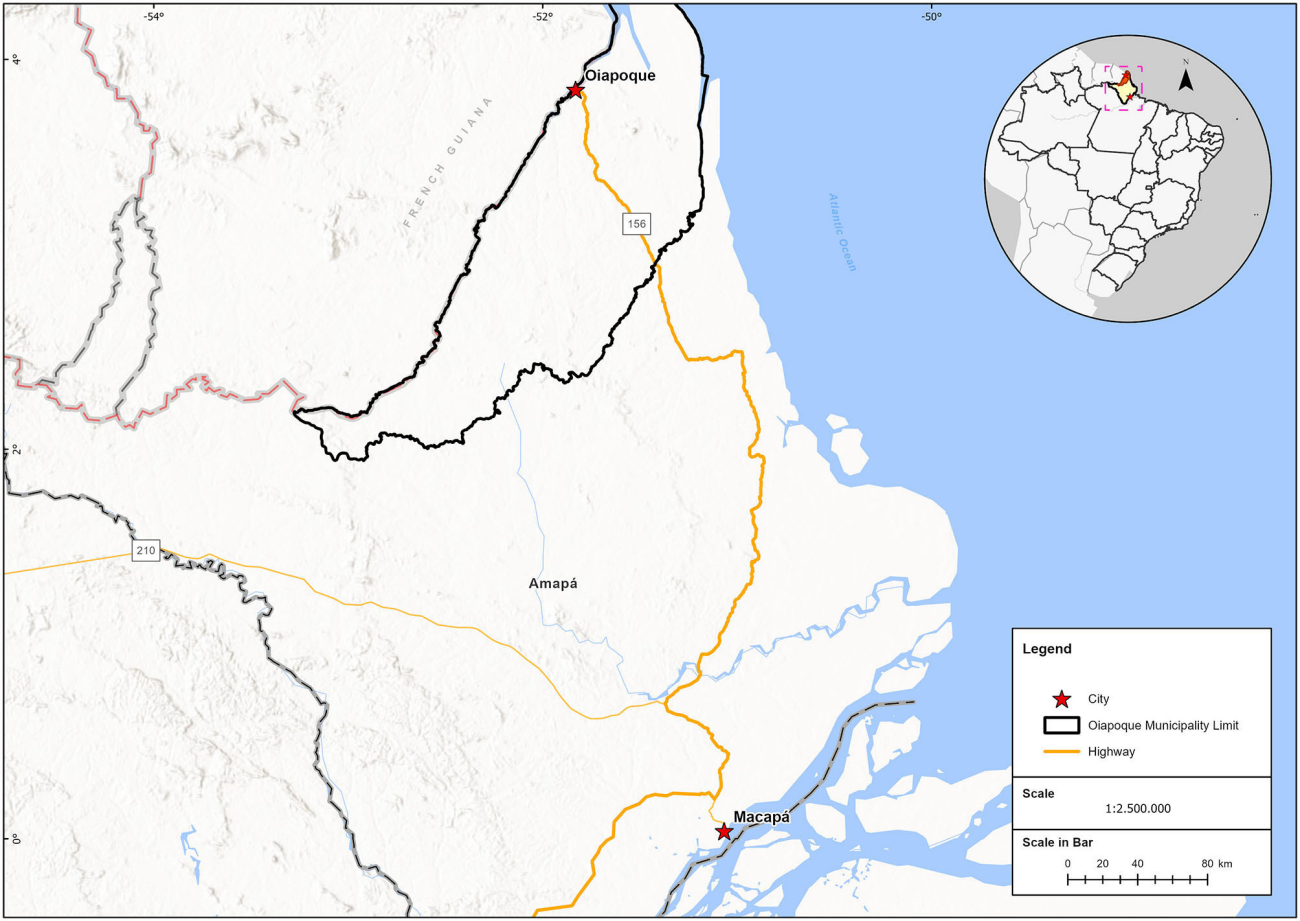
Location of the state of Amapa Brazil, and the municipality of Oiapoque. Border with the French territory, French Guyana.

Within the northern region, Oiapoque is a Brazilian municipality in the state of Amapá and the only one to share borders with a European overseas territory—French Guiana (5) (Figure 1). This unique municipality blends a number of existing dynamics, namely, migratory, economic, social inequalities, linguistic diversity, social and demographic isolation, and political issues (5–7). Oiapoque is located 560 km from Macapá, the capital of the state of Amapá. The municipality of Oiapoque has five free basic health units, a LAFRON border laboratory, and a state hospital. Until 2017, Macapá was the referral center for the treatment and follow-up of persons living with HIV (8). Oiapoque and Macapá are separated by an average of 9 h, including a 160 km segment of a rough dirt road in the forest, which is extremely muddy during the rainy season, extending the duration of the trip considerably. Since the beginning of the HIV epidemic, this tiring, long, and expensive trip has been a huge barrier to accessing HIV care and follow-up. However, since 2019, Oiapoque has been offering care to people living with HIV due to a cross-border collaboration on public health policy between French Guiana and Brazil and a proactive approach by the Health Secretariat of Oiapoque (5). Thus, treatment and care became available at the Basic Health Unit Nova Esperança.

The research and interviews were developed in all five health units in Oiapoque. As of December 2020, the Nova Esperança Basic Health Unit counted 62 people living with HIV on treatment, with six lost to follow-up and five transfers. The extreme north of Brazil and the Oiapoque region have intense migration, and this population mobility complicates patient follow-up and can be a source of HIV introduction and dissemination (9). A cross-border territory with intense mobility requires adapted strategies for testing, linkage, adherence, and monitoring of treatment. In such circumstances, the cascade of care represents a constant challenge that requires research to identify the major insufficiencies in the system. In this context, our objective was to describe the cascade of care, as recommended by the Brazilian Ministry of Health, using a qualitative approach in the municipality of Oiapoque, to understand all the steps, from testing to continuous follow-up, patient retention, adherence to treatment, and reestablishment of patient follow-up for those who had interrupted care, as well as to analyze the challenges and vulnerabilities in the whole process.

## 2. Materials and methods

### 2.1. Study type

The study was exploratory and qualitative (10–12). It involved mapping of health structures in the study area of the research: Oiapoque-AP; interviews were conducted with health managers in Oiapoque, both at the municipal and state levels and with professionals responsible for basic municipal units and the border-LAFRON laboratory.

**Figure 2:**
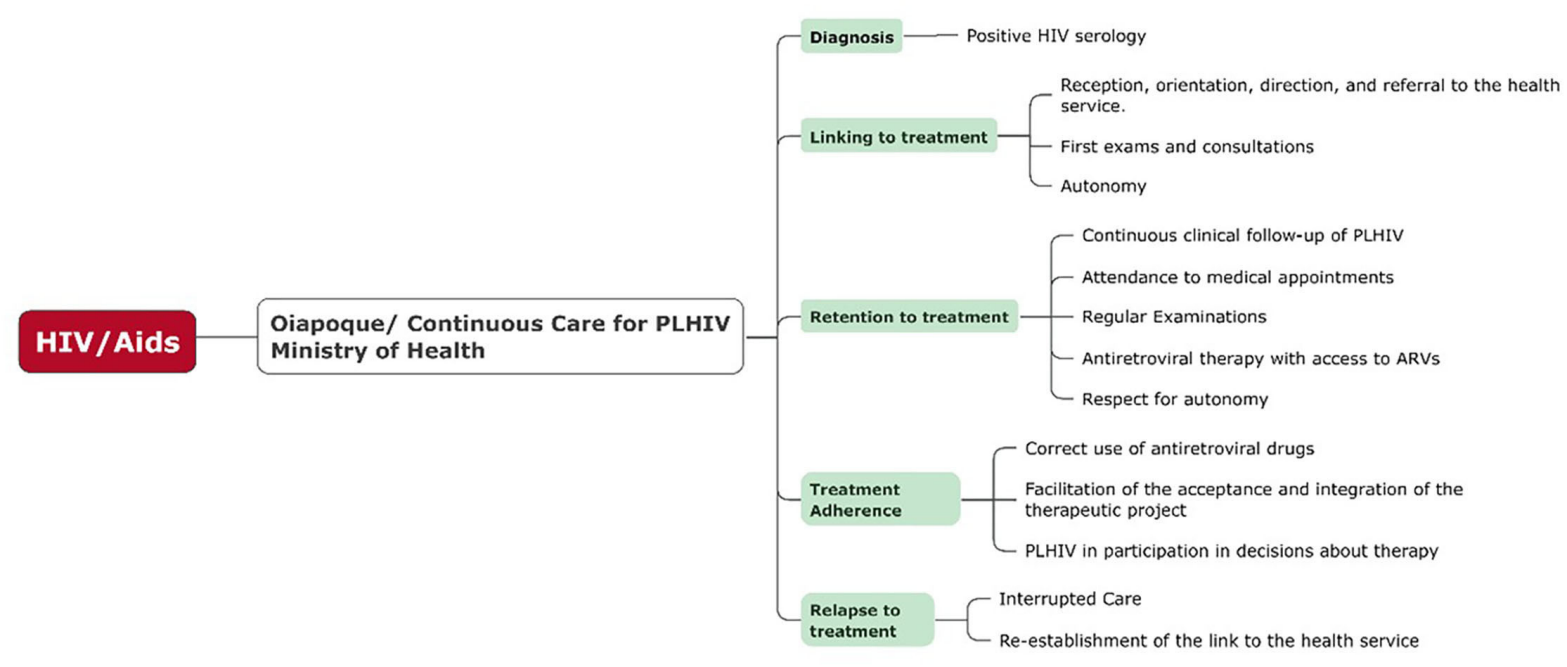
Required course of continuous care for people living with HIV (PLHIV) as recommended by the Ministry of Health, from diagnosis to continued treatment. Source: Own preparation based on the descriptions of the Manual of Combined Prevention of HIV–Ministério da saúde (1).

The continuum of care was used as a reference for healthcare professionals, workers, and managers. An organizational chart containing all the steps of the continuum—attachment, retention, adherence, and reattachment of patients with treatment interruption—served as a guide (“check list”) for analyzing access to services in the study area (Figure 2).

### 2.2. Mapping

The health units that make up the network of health services focusing on STIs/HIV and AIDS in the city of Oiapoque in 2020 were mapped and georeferenced using the GPS application “Gaia GPS.” In addition, we calculated the shortest route by car (time and distance) between the health units that performed the testing and the health units responsible for the treatment (Table 1). The geoprocessing program ArcGIS Pro 2.8 was used to draw up the flow map (testing units × treatment unit). Health structures involved in different stages of testing and treatment of HIV and AIDS in Oiapoque were surveyed in 2020.

**Table 1:** Detailed flows of the shortest route by car (time and distance) between the health units that performed the testing and the health units responsible for the treatment.

**Source**	**Destination**	**Distance (km)**	**Medium time**	**Cost of transportation**
UBS Vila Vitória	UBS Nova Esperança	9 km	20 min	R$ 40,00
UBS Julieta Palmeri	UBS Nova Esperança	1 km	4 min	R$ 20,00
UBS Planalto	UBS Nova Esperança	1 km	4 min	R$ 20,00
UBS Infraero	UBS Nova Esperança	3 km	5 min	R$ 20,00
Hospital Estadual de Oiapoque	UBS Nova Esperança	1 km	5 min	R$ 20,00
Vila Brasil (Posto de Saúde)	UBS Nova Esperança	110 km	4 h and 25 min	R$ 200,00

### 2.3. Interviews

A script with guiding topics was constructed, anchored on the central research questions, and administered through semi-structured interviews. The selection of interviewees for this qualitative analysis was done on a non-random, but purposeful basis, with all interviewees being representative and relevant to the research, that is, the importance of diverse sources. The first author (FD) conducted interviews with all professionals responsible for healthcare in the municipality of Oiapoque, Amapá State. As the study was exhaustive, there were no saturation issues (13).

The structure of the guide script was anchored on the central research questions to obtain information about the following: function of the healthcare professional in the municipality, current health situation and access to health in the municipality of Oiapoque, specialized care and treatment service, testing and linkage, presence or absence of educational actions and dissemination, care for migrants, presence of difficulties in the health unit, notifications, and records (14).

Professionals from the five basic health units in the municipality were interviewed: Basic Health Unit UBS Planalto, Basic Health Unit UBS Infraero, Basic Health Unit UBS Julieta Palmeri, Basic Health Unit UBS Vila Vitoria, and Basic Health Unit UBS Nova Esperança. Interviews were also conducted with managers responsible for the Frontier Laboratory-LAFRON, the State Hospital of Oiapoque, and the Health Secretariat.

It was not possible to conduct interviews in the health center of Vila Brasil because it was not functioning at the time of the study. However, an interview was conducted with a former health center worker and a resident of Vila Brasil to complete the analysis.

A total of 12 interviews were conducted from 1 October to 13 October 2020, by the study's first author (FD) and in the local language, Brazilian Portuguese. The group of healthcare professionals participating in the study was made up of seven nurses, five of whom were new graduates, one was a doctor, one was a pharmacist, one was a laboratory technician, one was a microscopist, and one was a nursing technician (Table 2). All interviews were recorded with signed consent and were conducted at the health units themselves, at a time chosen by the interviewees according to their availability.

**Table 2:** Age, nationality, and profession of healthcare professionals participating in the scientific research.

**Professional participant**	**Age**	**Nationality**	**Occupation**
Professional01	32	Oiapoque AP Brazil	Nurse
Professional02	31	Macapa AP Brazil	Nurse
Professional03	33	Belem PA Brazil	Nurse
Professional04	24	Oiapoque AP Brazil	Nurse
Professional05	35	Oiapoque AP Brazil	Nurse
Professional06	30	Oiapoque AP Brazil	Nurse
Professional07	36	Calçoene AP Brazil	Microscopist
Professional08	29	Oiapoque AP Brazil	Nurse
Professional09	33	Almeirim PA Brazil	Doctor
Professional10	29	Oiapoque AP Brazil	Pharmacist
Professional11	34	Macapa AP Brazil	Laboratory technician
Professional12	59	Oiapoque AP Brazil	Nursing technician

All interviews were recorded and transcribed completely and then analyzed and categorized according to the steps defined by content analysis. All relevant speeches to the research questions were transferred to a table where it was possible to differentiate the speeches of each participant interviewed (rows) and their relationship to each stage of care and treatment of HIV recommended by the Ministry of Health (column). The table design allowed us to identify the patterns of regularity and to analyze the ongoing process of care for persons living with HIV.

### 2.4. Ethical aspects

In carrying out the study, the ethical precepts of research involving human beings were respected, with approval of the project by the Research Ethics Committee of the Fundação Oswaldo Cruz (CAAE 29177120.8.0000.5248). To preserve the anonymity of the participants and maintain confidentiality, first names were replaced by fictitious names, a code was adopted for healthcare professionals (Saude1, Saude2, etc.), and their place of work was not mentioned in the text.

## 3. Results and discussion

A total of five basic health units, one hospital, and one health center were georeferenced in Oiapoque. These are the seven health units responsible for the execution of the HIV and AIDS care protocol in the city of Oiapoque–AP.

After visiting each of these health units, it was found that only the UBS Nova Esperança unit was responsible for the treatment/follow-up of persons with HIV and AIDS. The remaining units were responsible for testing and referral to the UBS Nova Esperança unit, referred to by healthcare professionals as “First Line.”

**Figure 3:**
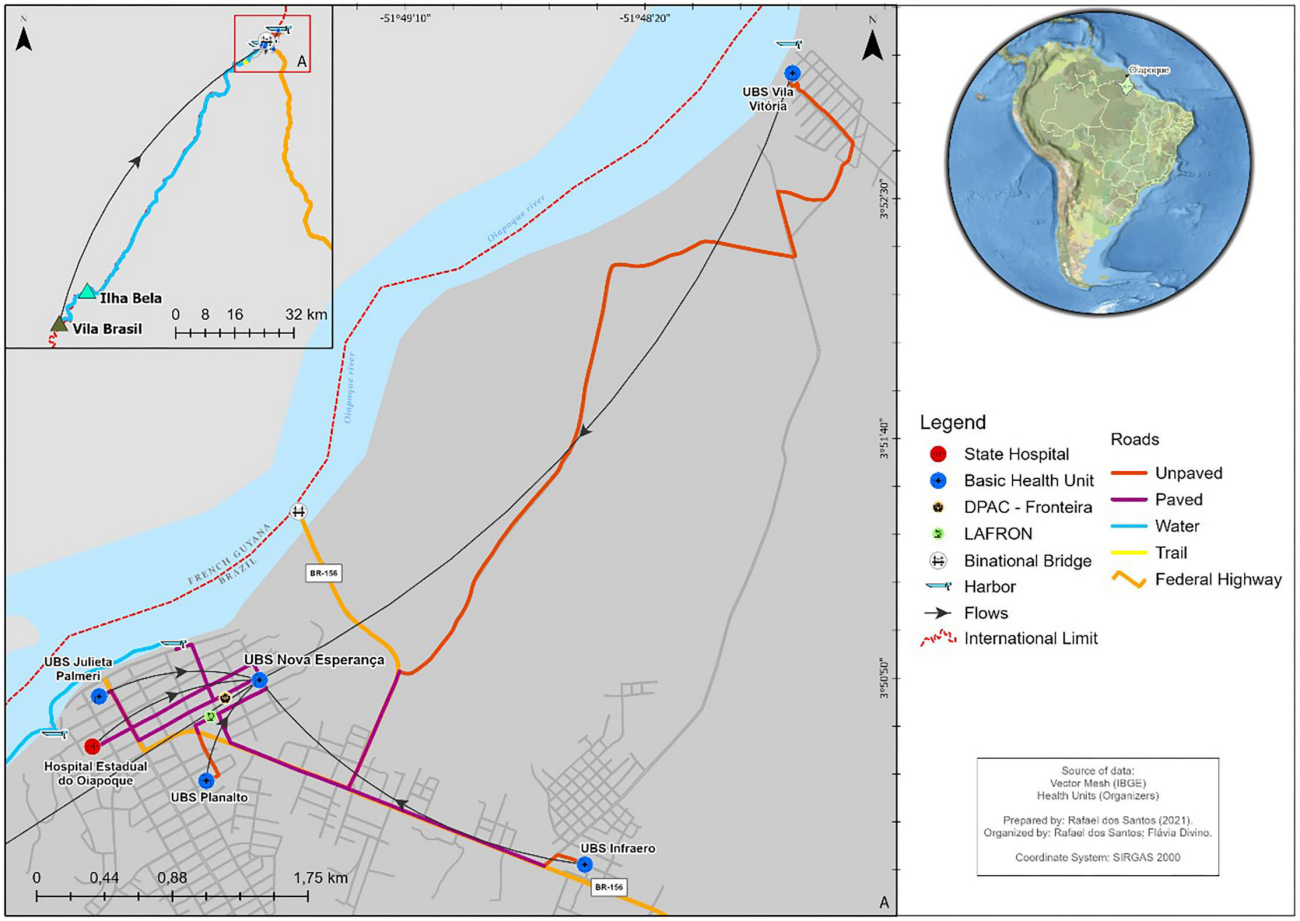
Routes between the units responsible for the referral and the unit responsible for the treatment.

The analysis of the routes between the units responsible for the referral and the treatment of HIV showed that the longest distances and difficulties with the route occurred in the Health Post of Vila Brazil and in the UBS Vila Vitória (Figure 3).

Between the Health Post of Vila Brasil and the Nova Esperança UBS, there is a distance of approximately 110 km, a route that largely requires a “catraia” (boat), followed by a small part where the patient must walk (on foot) and, when arriving in the city, has the possibility to use a private car or travel on foot to the unit responsible for the treatment. It takes an average of 4 h and 25 min to complete this journey (Table 1).

The second-most challenging travel situation (between UBS Vila Vitória and UBS Nova Esperança), despite having a much shorter distance and time compared to the previous situation should be treated as a situation that can vary in relation to the average travel time because much of its path occurs on an unpaved road that may become impassable during times of heavy rain.

In terms of travel from other health units mapped, patients had no major difficulties getting to the unit that provides the treatment. However, for all units, patients must walk or use a car (personal or cab) or any other private vehicle because the city offers no low-cost public transportation.

### 3.1. Diagnosis

Of the 12 interviews conducted, all the people in charge of all the basic health units confirmed the functioning of the testing service in the municipality:

“Look, the patient he will do the test right. If he is diagnosed through rapid testing in the other UBS, he will be referred to the first line.” (professional03)

The rapid tests used in Brazil, and therefore, in the study area, are simple immunoassays with results obtained in up to 30 min. They are performed in person, preferably in a non-laboratory setting, with whole blood samples obtained by digital puncture or oral fluid samples. They are completely confidential and done in the presence of a healthcare professional (15, 16). If the result of the first HIV test is reactive, a new test from a different manufacturer is required to confirm the diagnosis. However, it was observed during the research that there is a frequent lack of tests to confirm the diagnosis in the municipality of Oiapoque, and this is a point that deserves the attention of the state of Amapa, since there is a report that there has already been a case of false positive previously diagnosed in a local UBS:

“The test...the rapid HIV test works in the UBS, in all the posts. We have the rapid test here precisely to do a counter test and to check if the rapid tests are being done correctly in the basic units. Because one year I had a failure. A patient was given a false positive, the nurse called me, I did the test again for this patient and it was negative. You see, just for this one patient that we were able to identify an error, it was already worth the idea that we had of doing this monitoring.” (professional02)

In two moments in the interviews, there was also a report about the lack of the necessary tests for the confirmation of the diagnosis:

“So, we have to do another one which is the retest right. Although the test that we are doing here for HIV is the counter test because we do not have the other type of test. The retest is the only one being made available to us.” (professional10)

“Because there is the Biomanguinhos, right, which is the counter test, and there is the Bioeasy, which is the other one, if I am not mistaken. We are not having the Bioeasy, we are only doing the counter test, that is available in all units.” (professional10)

Even from the mapping done in the study (Figure 3), we can see that diagnosis is a widely distributed service in Oiapoque: all units provide it to the population. However, it is vital to pay attention to this point so that positive tests are reliably confirmed.

### 3.2. Linking

If the diagnosis is made in the unit qualified for this and the course recommended by the Ministry of Health is followed with a reactive result, the next step is to refer the patient to the first line of care and then start the free and appropriate treatment offered by the Unified Health System (15, 16). However, participant interviews revealed that referring a patient to a specialized service is not a trivial task. It is known that referral is necessary, but it is not clear how to do it. In addition, it is not clear how to minimize problems and make referrals in the most effective manner for the patient.

The municipality lacks consensus regarding where to refer patients, who should refer them, how to refer them, and under what conditions they will be referred. In one of the interviews, the first line of care is not even mentioned, demonstrating that there is no clear and unequivocal chain/sequence of information in the municipality, thus preventing the patient's access to what is legitimately offered, providing attention and integral healthcare in a free and universal way, and consequently aiming at his/her wellbeing:

“We have the rapid tests, only the rapid tests understand...here in the hospital. We do the rapid tests in patients requested through medical prescription. The doctor requests and does them, all the pregnant women as well. When a pregnant woman comes in, we do the tests that are already a protocol of the MH, we do the rapid test, and once the test is positive, we notify them, we notify them, we forward them to our documents sector and then they send them to the municipality's epidemiology department.” (professional01)

However, we can confirm that the first line in the municipality was a recent service and of extreme importance for Oiapoque:

“And the nurse who does all the consultations and follows this patient more closely, a more humanized treatment. A treatment that is new in the city, it's a new job that has only been around for two years... that has pleased this expectation, it has made it much easier because there are patients who can't afford to go to Macapá.” (professional04)

Linking is a process that requires welcoming, orientation, and direction for someone who has just been diagnosed and will need continuous care from that moment forward. This process of orientation and the beginning of care need attention because it is the patient's first step in the therapeutic process (16). What we noticed is that there is a consensus in most basic units that it is necessary to refer this patient to the first line. Unfortunately, there were no answers that incorporated this whole complex process. They were straightforward and very direct answers:

“If positive, we notify and send to the first line.” (professional06)

In this case, we can see the importance of this process given that, if the patient is not informed and calm and has an adequate understanding, they run the risk of not returning and not even starting treatment in the city. In one of the interviews, we noticed this concern with the reception. During another visit, the staff explained how things worked to the patient when they felt the need to do so. Explaining things to a patient has long been a concern of healthcare professionals themselves, who often feel unprepared to give information to the patient at a moment that they understand to be difficult:

“We don't have a structure that will allow us to follow up with him, here we only have testing. We also have to have a space for welcoming patients. Understand?” (professional08)

“If the patient tests positive, we send him to the first line. In other words, this patient is already in another sector, so we transfer him there. As for how is it done, many times we have to talk to them. The nurse himself has to talk to the patient about his results, because if I refer this patient, he will want to know why he is being referred. Because when the patient comes to do the rapid test, he already comes with his psychology shaken, right? He already comes a little afraid. So, if you say that he has to move to another sector, he instinctively knows that something is not right. So, we try to talk to this patient, say “look, there was an alteration in your exam.” (professional10)

It is pertinent to mention that in all the interviews carried out, no mention of autonomy for self-care was found or observed in the dialog, even though an emphasis on care for this new patient was demonstrated:

“In the first line he is attended, he is going to have his reception, he is going to be referred for a consultation with the doctor. He will also be directed to do a laboratory test to confirm, and after the results are in, the prescription will be made, and we will initially start with the preferential cocktail, where a month of treatment is distributed, you know. Aiming at the probability of this patient presenting a reaction to the medication, or something like that, since there is the issue of his adaptation and also to know if this medication is really going to have any effect on him. Then, for a month, we follow up, trying to know if the treatment is really working, in case this treatment does not cause any adverse reaction, any side effect in the patient, if he adapts well and we see that it is... it is going to influence his viral load, then we start to spread it to two months, three months, and this patient is continuously followed up. We schedule appointments for him to return, to verify this situation, and the dates for him to be tested. And so, we continue.” (professional03)

Adherence is also a collaborative process where adherence, acceptance, and integration of the person living with HIV into a therapeutic project are sought. It is necessary to pay attention and respect autonomy for self-care. As Oiapoque is a border municipality and has a fluctuating population, there was a presence of migrants and miners in the group of patients registered in the first line of care; thus, it is important to listen to them so that they can effectively participate in the treatment. The main strategy and goal of properly performed ART was to reduce the viral load circulating in a given society. This will reduce the rate of new infections. For this to happen, people in treatment need to reach an undetectable viral load, and this will only be possible with everyone's collaboration and commitment.

### 3.3. Retention and adherence

The moment of retention is a clinical follow-up, the existence of regular examinations, and access to antiretrovirals (ARVs). The Ministry of Health defines adherence as the correct use of prescribed drugs based on doses, schedules, and other indications. In these two moments of the process, the absence of an infectious disease specialist was observed. This fact was observed in all basic units and sometimes mentioned during the interview. Furthermore, the need to refer patients to a specialist, which is a known problem in Oiapoque, and the distance and difficulties of displacement and transportation were mentioned:

“We don't have, we have general practitioners, only.” (professional09)

“Well, our doctor refers to Macapá, that...there for the SAE, only there is another issue, not all patients can go.” (professional09)

Observed issues included the lack of routine consultations, which hinders clinical follow-up and makes abandonment inevitable. It was also very recurrent in the interviews and dialogues to observe quotes about gold miner patients, their frequent long trips, and the presence of these workers within the group in continuous treatment:

“We have difficulty with those who go right here in the city, our difficulties are here, they don't leave here, they are the ones that give more headache I say so... because I think they settle down, they don't want to come and we have to seek to know what happened, sometimes they come here with a lot of difficulty, but then they disappear afterwards.” (professional09)

“No, well... there are some miners, couples... they all go to the mine, they spend some time there. Before all this happened, they were here, they hadn't left, but since they had the opportunity, they left now.” (professional09)

“I believe that about 20 are garimpeiros.” (professional09)

For regular consultations, continuous clinical follow-ups and the adequate linking of the patient to the health service and regular examinations are also necessary. Unfortunately, this is still a point of dependence between the municipality and Macapá, even with the long trip, extensive route, and existing difficulties:

“It is... what we used to do was the load only that since March we had “n n” problems with the Genesis expert, mainly the electrical part that we are having variations of the electrical part of the laboratory, I asked for an adequacy of a re-evaluation of this electrical part of the border laboratory, so we could be reconnecting the equipment.” (professional02)

“We are sending both viral load and CD4 to Lacen in Macapá.” (professional02)

Even with such difficulties during the course of continuous treatment, we observed in interviews the access to medication and this process is accompanied by the professionals responsible for dispensing:

“Is dolutegravir, lamivudine, and tenofovir. Tenofovir plus lamivudine is two in one...one pill only that has these two active ingredients. And dolutegravir is another drug. So, the patient will take two pills a day.” (professional03)

“Yes, yes. In the case, at the time of dispensing I do the dispensing of these patients, is where I do a certain consultation, I talk to them. I check, I ask several questions about the issue of their day-to-day life, food, exercise. I do counseling, ask about the medication, if they are feeling anything, any kind of pain, nausea, insomnia, how they take this medication, schedule.” (professional03)

### 3.4. Relapse or return to treatment

“Working with HIV patients is very complicated because it is as if they were children that we have to always be there for them to try to do the right thing and make them follow the treatment because many want to give up, and that is the worst moment, it is knowing that we are doing the hard work and knowing that at any moment one of them wants to drop everything and we have to talk to them to try to make them understand that this is not the way it is necessary for them to follow the treatment, this is very complicated.” (professional09)

**Figure 4:**
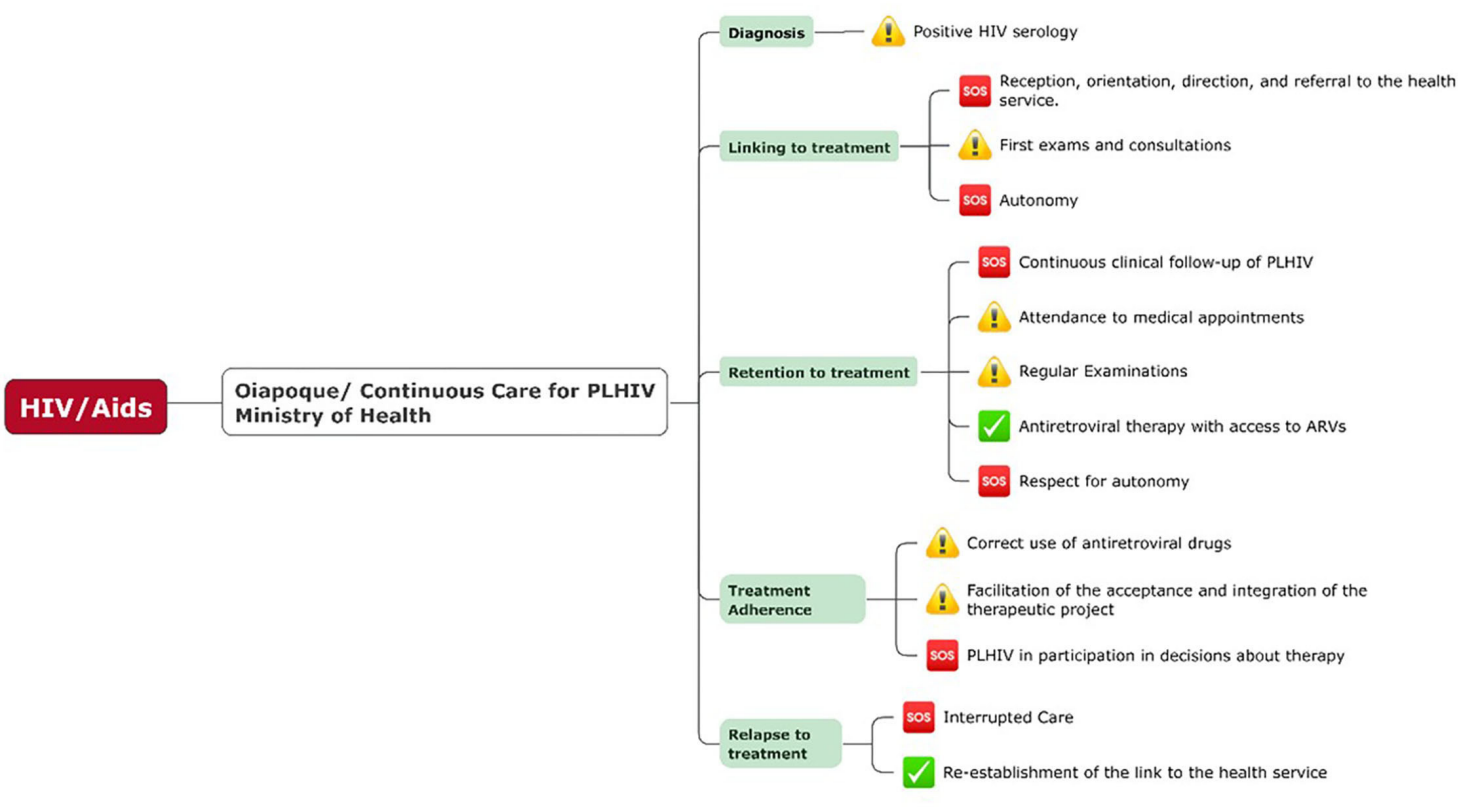
The necessary pathway of continuous care for people living with HIV (PLHIV) recommended by the Ministry of Health, from diagnosis to continuous treatment, marked with symbols of attention, S.O.S, and “check” in green, referring to the points that need attention and care, as well as the points that the service has a good resolution. Source: Own preparation based on the descriptions of the Manual of Combined Prevention of HIV–Ministério da saúde (1) based on the construction of the results and analysis carried out in Oiapoque.

Figure 4 shows the elements of the continuum of care that need attention in their functioning in the health service; a red SOS symbol marked the elements where a greater difficulty in the treatment scheme was observed, yellow marked the elements that needed attention, and green marked the elements that fell well within the treatment path.

## 4. Conclusion

Brazil is often presented as a model country for its fight against HIV and its effort to sustain an optimal cascade of care. However, despite its universal health system, this huge and socioeconomically heterogeneous country also has very heterogeneous cascades of care. Thus, in Oiapoque, an isolated town in northern Brazil, local access to ART only became available in 2017—nearly 30 years after urban Brazil. The arrival of ARV distribution in Oiapoque was thus an event that forced health structures to integrate this new option (before patients had to travel 579 km) and reorganize to coordinate health structures in this new landscape. This study is the first description of a “new” organization in Oiapoque, which has not had much time to settle into an optimized organization as in other more populated parts of Brazil.

Here, using qualitative interviews with healthcare professionals working in structures that provide HIV testing or treatment services, we reported the vulnerabilities in the continuum of care cascade. When Oiapoque implemented the first line of care delivery, these processes required attention and efforts to optimize. By understanding this continuum of care, we can provide knowledge to healthcare professionals and rethink strategies for action and work. This is both in relation to the first line of care but also across all healthcare systems given that the cascade of care is based on the coordination of efforts.

Many barriers were present. The lack of confirmatory diagnostic tests, psychologists, training and continuous education of healthcare workers, communication and information between the basic health units regarding referrals and basic information, difficulty listening to patients, how to provide therapeutic education to support autonomy, and interruptions in follow-up are all obstacles to the HIV care cascade in Oiapoque.

Better public policies can be guided by these aspects. New decisions taken by the health secretariat that promote patient autonomy and a better understanding of the difficulties facing the municipality are sought. These solutions seek solutions not only for the performance of professionals but also for improving care for patients.

This country has a single size and shape, but it can be said that there are different “Brasis” within Brazil. Although the Ministry of Health recommends a treatment that is perfectly designed, structured, and adapted to local communities and cultures, it is acknowledged that it is crucial to know and understand the local society in order to optimize prevention, diagnosis, and care.

Previously identified knowledge and beliefs are important so that the necessary additional information can be offered, promoting active listening and identifying the patient's expectations even before starting treatment and, thus, being able to build a therapeutic scheme together. Although trained psychologists are often rare human resources in this remote area, psychological support to assist the user and the family and social support are essential, and accurate information on the different stages of the cascade of care should be provided.

These findings originate from a small border town in northern Brazil and it could be argued that they are only of local relevance. However, what is reported here in fact applies to many other situations in Brazil and Latin America. Indeed, isolated towns with mobile populations and scarce human resources are a pattern that is broadly observed throughout the Amazon and further along the continent. A review on the continuum of care in Latin America concluded that the healthcare systems are deficient in the continuum's different stages and that, in some cases, only a small proportion of individuals achieved the desired outcome of virological suppression (17). The review also remarked that data for most Latin American countries were often insufficient to build reliable metrics. Hence, many people living with HIV in Latin America remain unaware of their status, are diagnosed late, and enter into care late. The main determinants for late diagnosis and failure to be linked to and retained in care are stigma, administrative barriers, and economic limitations. The review concluded that policymakers needed reliable data to optimize the HIV care continuum in Latin America.

We thus believe that the present findings can be useful for the public health network. The participation of healthcare professionals in this study emphasizes the need for joint constructions. The participation and empowerment of the community are fundamental for improving the actions of the municipality and the strategies of the single health system SUS, notably with regard to the HIV epidemic, in these border regions.

## Data availability statement

The raw data supporting the conclusions of this article will be made available by the authors, without undue reservation.

## Ethics statement

The studies involving human participants were reviewed and approved by Research Ethics Committee of the Fundação Oswaldo Cruz (CAAE 29177120.8.0000.5248). The patients/participants provided their written informed consent to participate in this study.

## Author contributions

FD and PP conceived and designed the research. FD and RP conduction of the research. FD, MN, and PP analyzed the data and wrote the paper. All authors contributed to the article and approved the submitted version.
